# Effects of Therapeutic Antibodies on Gene and Protein Signatures in Asthma Patients: A Comparative Systematic Review

**DOI:** 10.3390/biomedicines10020293

**Published:** 2022-01-27

**Authors:** Maria J. Martin, Miguel Estravís, Asunción García-Sánchez, Jacqueline Pérez-Pazos, María Isidoro-García, Ignacio Dávila, Catalina Sanz

**Affiliations:** 1 Instituto de Investigación Biomédica de Salamanca (IBSAL), 37007 Salamanca, Spain; mjmartinma@saludcastillayleon.es (M.J.M.); estravis@usal.es (M.E.); jperezpaz.ibsal@saludcastillayleon.es (J.P.-P.); misidoro@saludcastillayleon.es (M.I.-G.); idg@usal.es (I.D.); catsof@usal.es (C.S.); 2 Red Cooperativa de Investigación en Salud–RETICS ARADyAL, 37007 Salamanca, Spain; 3 Departamento de Bioquímica y Biología Molecular, Universidad de Salamanca, 37007 Salamanca, Spain; 4 Departamento de Ciencias Biomédicas y del Diagnóstico, Universidad de Salamanca, 37007 Salamanca, Spain; 5 Departamento de Medicina, Universidad de Salamanca, 37007 Salamanca, Spain; 6 Servicio de Bioquímica Clínica, Complejo Asistencial Universitario de Salamanca, 37007 Salamanca, Spain; 7 Servicio de Inmunoalergia, Complejo Asistencial Universitario de Salamanca, 37007 Salamanca, Spain; 8 Departamento de Microbiología y Genética, Universidad de Salamanca, 37007 Salamanca, Spain

**Keywords:** therapeutic antibody, mepolizumab, benralizumab, omalizumab, asthma, transcriptome, proteome

## Abstract

Several biologic therapies that target inflammatory modulators are now used for treating patients with uncontrolled, severe asthma. Knowledge about how this type of treatment modifies the molecular milieu is rapidly increasing. Thus, this systematic review aimed to compile the reported effects of therapeutic antibodies on the transcriptome or proteome of asthma patients. Studies of asthmatic patients under biological treatment describing transcriptomic or proteomic changes upon treatment were included. Preclinical or single gene/protein studies were not considered. PubMed and Scopus search was performed in August and September 2021. Following PRISMA guidelines and GRADE recommendations, we selected 12 studies on gene or protein expression changes in patients treated with the antibodies currently approved by EMA and the FDA. All studies were at low risk of bias as per the RoB2 tool. Different gene clusters have been identified to change upon omalizumab treatment, found a reduction in eosinophil-associated gene signatures after benralizumab treatment, and protein profiles were different in patients treated with mepolizumab and in those treated with benralizumab. The main potential biomarkers proposed by the selected studies are shown. These results may contribute to discovering biomarkers of response and selecting the best therapy for each patient.

## 1. Introduction

During the last few decades, asthma and related diseases have become a global health problem affecting all age groups. The high incidence of asthma in the population of some countries suppose a burden to health care systems and loss of productivity and quality of life. Asthma is a heterogeneous disease, usually characterized by chronic airway inflammation. It is defined by the history of respiratory symptoms, such as wheezing, shortness of breath, chest tightness, and coughing, which vary over time and in intensity, together with variable expiratory airflow limitation [1].

Asthma has been classified as either a T2-type and a non-T2-type [2]. T2-type asthma presents a T2-type immune response, characterized by Th2 cell-driven inflammation and mainly includes allergic asthma, late-onset eosinophilic asthma, and aspirin-exacerbated respiratory disease (AERD) [3]. On the other hand, non-T2-type asthma refers to asthma without a T2-type immune response, with Th1 or Th17 cell-driven inflammatory responses, including neutrophilic asthma and smooth muscle mediated paucigranulocitic asthma [3].

Current treatments for asthma aim to control symptoms and reduce the risk of future exacerbations. Nevertheless, some asthmatic patients have severe asthma with persistent symptoms, reduced lung function, or multiple exacerbations despite maximal treatment [4]. 

Over the last years, several monoclonal antibodies targeting specific inflammatory pathways have been developed and approved to tackle this problem and improve the patients’ quality of life [5]. These monoclonal antibodies block IL-5 cells, such as mepolizumab, reslizumab [6], IL-5 receptor (IL5-Rα), i.e., benralizumab [7], and IL-4 and IL-13 via IL-4/IL-13 receptor s(IL4-Rα), i.e., dupilumab [8], abrogating their inflammatory signaling pathways in allergic eosinophilic asthma. Omalizumab, which blocks IgE, has also shown efficacy in the treatment of severe allergic asthma [9].

Although biological agents are revolutionizing the management of severe uncontrolled asthma, 13-31% of patients can be unresponsive [10,11,12,13]; in addition, there are currently no parameters to predict the individual response to any biologics. In this sense, there is a remarkable lack of pharmacogenetic biomarkers that allow a more precise and practical selection of patients and establish uniform treatment response criteria. Thus, further effort is needed to identify other potential molecular targets that could be used as prognostic and therapeutic biomarkers that will facilitate therapeutic strategies tailored to each patient’s requirements [14]. It also entails reducing unnecessary expenses in patients who would not obtain any benefit, which is particularly interesting, considering the high costs of biological drugs (upwards of thousands of euros per year).

The genetics of asthma appear to be quite intricate, involving multiple genes and epigenetic mechanisms, each with a small effect size [2]. Therefore, next-generation sequencing techniques may offer an excellent approach to shed light on the complex genetic networks underlying the different endotypes of the disease. This systematic review aimed to compile the publications on transcriptomics, proteomics, and epigenetics in asthmatic patients treated with biologic therapies.

## 2. Materials and Methods

This systematic review has been performed following PRISMA guidelines for Systematic Reviews and Meta-Analysis-2020 checklist [15] (see Supplementary Material) and GRADE recommendations [16]. Protocol was registered at PROSPERO (ID304691).

Original articles and meta-analyses indexed from January 2000 to August 2021 describing the effects of biologic therapy of asthmatic patients on gene expression were searched. We identified eligible studies using the following inclusion criteria: (1) primary study or meta-analysis; (2) written in English; (3) human subjects, both children and adults; (4) patients who had asthma and were under biological treatment; and (5) studies describing gene or protein expression changes upon treatment. The exclusion criteria were: (1) animal, in vitro, or in silico studies; (2) review articles; (3) single gene or protein studies; (4) articles focused on other diseases in which asthma was merely mentioned; and (5) studies lacking pre-treatment data or healthy controls to compare with. 

We performed the literature search between August and September 2021 in PubMed and Scopus databases, using the following terms: “asthma” AND “gene expression” OR “transcriptomics” OR “RNASeq” OR “proteomics” AND “benralizumab” OR “mepolizumab” OR “omalizumab” OR “dupilumab” OR “reslizumab”. We omitted “biologic therapy” or “antibody treatment”, since the ambiguity of such terms retrieved many irrelevant articles. 

We sought out the effects of biologics therapy on gene expression or protein expression at an “omics” level, such as transcriptomics, genome-wide association studies (GWAS), or proteomics. Those studies lacking a comparison to baseline or referring to a single gene or protein were considered of limited relevance and excluded. 

Four authors individually reviewed the database search results, assessing titles, evaluating abstracts, and considering or not the study for full review. Any disagreements in either the title/abstract or the entire manuscript review phases were resolved by consensus. All eligible studies were formally evaluated and included in this systematic review. 

The authors independently graded the risk of bias of the included studies using the RoB2 tool [17] and evaluated the quality of evidence as per the appraisal form for Longitudinal Studies by the Evidence Evaluation Tools and Resources (LEGEND) from the Cincinnati Children’s Hospital form for Longitudinal Studies (https://www.cincinnatichildrens.org/research/divisions/j/anderson-center/evidence-based-care/legend; accessed on 1 November 2021). In this appraisal, a total score over nine was considered high quality evidence, a score between six and eight merited moderate quality evidence, and low quality was attributed to studies under a score of five.

## 3. Results

### 3.1. Selection, Bias and Quality of Articles

**Figure 1:**
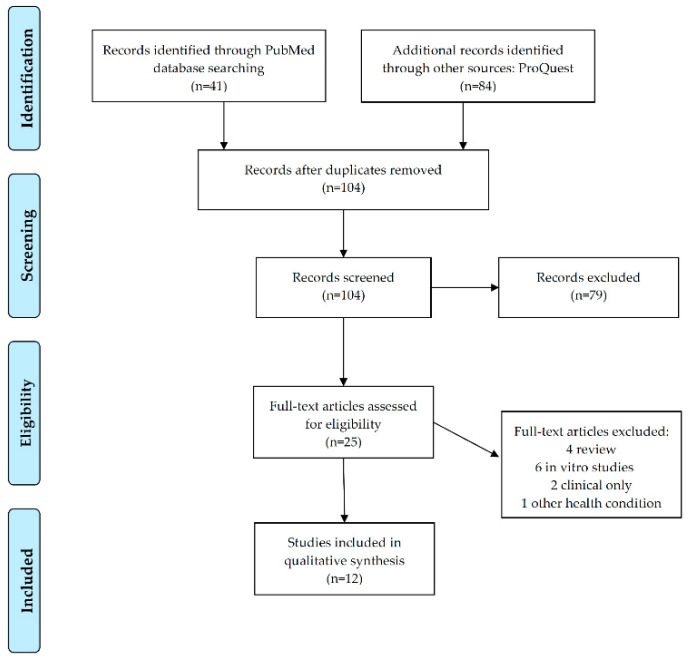
PRISMA-based flow diagram of the selection process (www.prisma-statement.org; accessed on 1 September 2021).

The database search yielded 104 articles after duplicate removal (Figure 1). After title and abstract review, 79 articles were excluded since they did not fulfill eligibility criteria, i.e., in vitro/animal studies, literature reviews, articles focused on other conditions that merely cited asthma, and studies including only clinical data. As a result, 25 articles qualified for full-text review. Of those, 13 studies were further eliminated since they were review articles, referred to in vitro data exclusively, or focused on aspects other than gene expression. The flow diagram of the selection process is shown in Figure 1.

Therefore, 12 articles qualified for the qualitative synthesis, two including omalizumab treatment [18,19], two referring to mepolizumab [20,21], four reporting data on benralizumab treatment [22,23,24,25], two comparing mepolizumab and benralizumab treatments [26,27], one comparing reslizumab and mepolizumab [28], and one about fekanizumab [29]. A summary of the selected studies is presented in Table 1. 

We followed the Cochrane guidelines to assess the risk of bias of the selected studies, using an adapted version of the RoB2 tool to fit the specific nature of the studies. The tool evaluates the randomization process, deviation from intended intervention, missing outcome data, measurement of the outcome, and selection of the reported result. An in-depth appraisal of each article did not find any concerns regarding these topics; thus, all the selected studies qualified as having a low risk of bias (Table 2).

Concerning the quality of evidence, 3 out of the 12 articles were considered moderate quality articles due to methodology validity concerns and sample size limitations (<20 patients). Also, it is worth mentioning that five studies included a conflict of interest of the authors (Table 3).

### 3.2. Omalizumab

Upchurch et al. [18] published an expression profiling study on 45 patients with uncontrolled asthma under omalizumab treatment compared to 17 healthy controls (HC). They reported 34 patients as responders to omalizumab and 11 as non-responders and took samples at baseline and 6, 14, and 26 weeks of treatment. All data are publicly available at the GEO repository (GSE134544). 

When analyzing the data, the authors found that both responder and non-responder expression profiles were similar to HC during the first six weeks of treatment. Eight gene clusters were identified, including genes related to protein synthesis (cluster 1), T cell/NK cell/cytotoxicity (cluster 2), hematopoiesis (cluster 3), cell cycle control and proliferation (cluster 4), T cell regulation and activation (cluster 5), monocytes (cluster 6), glucose metabolism (cluster 7), and inflammation (cluster 8). Significant changes between responders and non-responders were found in clusters 2, 3, 7, and 8 at baseline; in clusters 2, 3, and 7 at 6 weeks; in clusters 3 and 7 at 14 weeks; and in cluster 8 at the final time point of 26 weeks. After modular analysis, the largest number of variations between asthmatic patients and HC occurred before treatment, and this difference slowly decreased upon omalizumab therapy in responders while non-responders showed a higher number of differentially expressed modules when compared with HC at week 26. Regarding pathway analysis, the 293 transcripts overexpressed in responders were related to Th2 and Th1 responses. The 496 transcripts under expressed in non-responders were connected to the suppression of inflammation, and other connections were associated with the promotion of allergic inflammation. In summary, responders showed increased immune cell motility while non-responders showed increased cytokine signaling and inflammation networks.

Using the same transcriptomics data, Zhang et al. [19] carried out a bioinformatics analysis to find potential biomarkers for predicting patient responses to omalizumab treatment. They identified ten modules using hierarchical clustering, the red cluster containing 547 genes, the closest to omalizumab responders (Pearson coefficient = 0.89; *p* = 5e-16). Within this module, only *CD3E* and *CD79A* had significantly higher expression in the responder group than in the non-responder group (*p* = 0.014 and 0.037, respectively), but only *CD3E* remained significant after logistic regression analysis. CD3E is part of the TCR-CD3 complex on the T-cell surface, crucial in T-cell development and activation.

Out of the articles listed in our systematic search, we decided to exclude the study by Hachim et al. [30]. The authors showed that the levels of expression of periostin (POSTN) in blood and saliva of severe asthmatic patients were lower in a group of patients treated with omalizumab than in those patients without treatment. Nevertheless, the patients were recruited when they were already under omalizumab treatment. 

### 3.3. Mepolizumab

Buchheit et al. [20] investigated how mepolizumab treatment improved respiratory inflammation in AERD patients. A group of 18 AERD patients receiving standard of care was compared with 18 received mepolizumab for at least three months. Different blood cell populations were analyzed by flow cytometry, and nasal epithelium mRNA expression was also investigated. Regarding gene expression, 242 genes were differentially regulated in subjects treated with mepolizumab. The 94 upregulated genes included *TJP3*, *ACTN4*, and *AMOT*, which are involved in tight junctions. Among the 148 downregulated genes, authors highlighted *CLDN17*, which is also related to tight junctions. CRTH2 surface expression was higher on blood cells of treated patients than on those from non-treated subjects, although eosinophils and basophils count decreased in the mepolizumab group.

Condreay et al. [21] tested the association of genetic markers that may predict responses to mepolizumab in two cohorts of severe asthma patients, i.e., DREAM and MENSA studies, including a total of 589 patients, 441 who received mepolizumab and the rest who received a placebo. They conducted candidate genetic variant and GWAS analyses, finding eight variants with weak evidence of association (*p* > 0.05). However, this association was driven mainly by a small subset of patients treated with the highest experimental dose. Thus, no pharmacogenetic effects were unambiguously detected in this article, and the authors recommended further and more extensive studies.

### 3.4. Benralizumab

We reviewed four articles focused on benralizumab therapy against asthma. Benralizumab is a monoclonal antibody that specifically binds the IL5Rα, producing antibody-dependent cell-mediated cytotoxicity by natural killer cells and inducing apoptosis of eosinophils.

Sridhar et al. [25] investigated the effects of benralizumab subcutaneous 100 mg every eight weeks on blood inflammatory markers through proteomic and gene expression analyses during two Phase II studies of patients with eosinophilic asthma. Results demonstrated that only two protein analytes, eotaxin-1 and eotaxin-2, were significantly upregulated following treatment with benralizumab in both asthma and chronic obstructive pulmonary disease (COPD), with higher levels in eosinophil-high patients than in eosinophil-low patients in both studies. Benralizumab was also associated with a significant reduction in the expression of genes related to eosinophils and basophils, such as *CLC*, *IL5RA*, and *PFSS33*; immune signaling complex genes (*FCER1A*); G-protein-coupled receptor genes (*HRH4*, *ADORA3*, *P2RY14*); and other immune-related genes (*ALOX15* and *OLIG2*). 

Another transcriptomic study of 41 patients with variable clinical responses to benralizumab focused on biomarkers related to responsiveness to treatment [24]. Gene expression analysis levels in peripheral blood were compared at baseline and after four months of therapy with benralizumab, showing significant reductions in the expression of 33 genes associated with eosinophilic inflammatory responses, such as *PTGDR2*, *ALOX15*, *IL5RA*, *SMPD3*, *CLC*, *HRH4*, *CYSLTR2*, and *RAB44*. On the contrary, 29 upregulated genes were related to neutrophils, such as serine hydrolase activity, neutrophilic degranulation, and neutrophilic activation. This analysis provided four distinct clusters in patients with severe eosinophilic asthma with variable responsiveness to benralizumab. 

Severe asthma patients can show a steroid-resistant asthma phenotype. Benralizumab reduces the oral corticosteroid dosage while maintaining control in severe asthmatics with peripheral eosinophilia [31]. To elucidate whether benralizumab modified corticosteroid sensitivity by suppressing type-2 inflammation, Hirai et al. [23] analyzed the gene expression changes on T cells from patients with severe asthma treated with benralizumab. The study demonstrated that treatment with benralizumab in patients with severe corticodependent asthma could restore the expression levels of key molecules involved in steroid response through the PI3K pathway inactivation. 

A transcriptomic study of 15 severe eosinophilic asthma patients treated with benralizumab was conducted to find new biomarkers as microRNAs that predict the response of benralizumab [22]. Serum miRNAs were analyzed before and after eight weeks of treatment, showing deregulation of miR-1246, miR-5100, and miR-338-3p in severe asthmatic patients after treatment, and suggesting that these miRNAs could be used as early response markers.

### 3.5. Mepolizumab and Benralizumab

A couple of studies by the same authors compared patients treated with mepolizumab and patients treated with benralizumab [26,27]. Both studies compared serum proteomic profiles from patients with severe eosinophilic asthma at baseline and after one month of treatment. 

In the first study [26], ten patients were treated with mepolizumab and eight with benralizumab. Four HCs were also included. The authors reported 38 differences among patient proteomic profiles. Two spots were exclusively found at baseline, while ten spots only appeared after one month of benralizumab treatment and five spots were only detected after one month of mepolizumab treatment. Benralizumab-treated patients showed increased plasmin, alpha-1-antitrypsin, plasminogen, alpha-2-macroglobulin, and ceruloplasmin levels, while mepolizumab patients showed increases in albumin, fibrinogen gamma, and factor B levels, among others. The most significant change related to benralizumab treatment was the increase of full-length ceruloplasmin, which was associated with lower serum oxidation levels in those patients.

Vantaggiato et al. [27] compared the serum proteomic profiles of patients with severe asthma before and after one month of treatment with mepolizumab or benralizumab since both treatments suppress IL-5 signaling pathways. In this preliminary study, the authors recruited five patients treated with benralizumab and five patients treated with mepolizumab. In addition to the molecular analysis, these patients were clinically evaluated after six months of therapy for lung function test parameters (FEV1, FEV1/FVC ratio) and asthma control test scores. Comparisons between before and after one month of treatment revealed a total of 22 differentially abundant spots corresponding to 17 protein species. Three proteins were significantly modified after both biological treatments: calcyphosin (CAYP1) was downregulated, and A1AT (alpha1-antitrypsin) and A2M (alpha-2-macroglobulin) were upregulated. In addition, different isoforms of ceruloplasmin were upregulated in patients treated with benralizumab, whereas haptoglobin was downregulated in patients treated with mepolizumab. These proteins emerge as potential biomarkers for therapy-induced responses and could be valuable to establish the most suitable biological treatment, i.e., mepolizumab or benralizumab, for a given patient.

### 3.6. Other Biologicals

Rial et al. studied serum miRNAs after anti-IL-5 biological treatment of severe asthma as possible response-biomarkers [28]. After eight weeks, sera of ten severe asthmatic patients treated with reslizumab and six patients treated with mepolizumab were analyzed. miR-338-3p, which is involved in essential pathways in asthma, such as MAPK and TFGβ signaling pathways, was dysregulated after treatment independently of the biological treatment. Authors concluded that miR-338-3p could be used as a biomarker of early response to reslizumab and mepolizumab in severe eosinophilic asthmatics and could be involved in airway remodeling and targeting genes related to MAPK and TGFB.

Badi et al. [29] proposed a different but interesting approach in their study. Taking advantage of a previously reported genetic signature of atopic dermatitis (AD) in patients who responded to anti-IL-22 (fezakinumab, FZ), they searched for such transcriptomic signatures in adults with severe asthma to determine whether they could be successfully treated with this biological. AD patients were classified as per their clinical response to FZ after 12 weeks of treatment, identifying those genes that changed significantly upon FZ treatment (FZ-DOWN). The FZ-DOWN signature included inflammation, Th2 response, and Th17/Th22 activation genes. Interestingly, the FZ-DOWN signature was also significantly enriched in the blood of severe asthmatics, mainly those with neutrophilic (adj.*p* = 0.0002) and mixed granulocytic asthma (adj.*p* = 0.0098) when compared with HC. Thus, the enrichment score of the FZ-DOWN signature in sputum of severe asthma patients was used for categorizing them into predicted-responders and predicted-non-responders to FZ. This approach could suggest that FZ might benefit T2-low severe neutrophilic asthmatics.

## 4. Discussion

In the present systematic review, we intended to gather all the published information about the effects of biological therapy on the gene and protein expression of asthmatic patients to identify potential biomarkers of response to treatment that could contribute to improving the management of severe asthma patients and selecting the best biological for each subject.

The first therapeutic antibody approved by FDA and EMA for persistent allergic asthma was the anti-IgE omalizumab (2003 and 2005, respectively). Anti-IL-5 monoclonal antibodies -mepolizumab and reslizumab- were approved by EMA for severe asthma with peripheral eosinophilia in 2015 and 2016, respectively. Dupilumab, an anti-IL4Rα, was approved in Europe in 2017 for atopic dermatitis and in 2019 for T2 asthma, while the anti-IL-5Rα benralizumab was approved for eosinophilic asthma in 2018 [32]. Therefore, their use for severe uncontrolled asthma is now usual, and many studies have been published regarding efficacy, safety, asthma control, and economic impact of all five biologicals in clinical settings [32,33,34].

Despite being widely used, few molecular studies have been conducted up to date in this field, and most of them refer to the expression of a specific gene or protein either in blood or airway tissues. Since our main goal was to seek biological markers of response to therapy, we limited our search to those articles using an ‘omics’ approach, such as RNASeq, transcriptomics, or proteomics. Being the therapeutic antibodies directed against crucial molecules, such as IL-4, IL-5, or IgE, it is expected that a plethora of genes and proteins rather than a single one would be affected by the therapeutic antibody. Thus, a gene/protein signature, including both up and downregulated species, would constitute a more accurate measurement of response to treatment.

Thus, we selected 12 articles about the effects of 1 or 2 of the approved biologicals on gene/protein expression. A summary of main outcomes is shown in Table 4. Most studies compared pre- and post-treatment expression, although we also found articles comparing asthmatic patients to healthy controls [26,27,29] or placebo versus biological treatment [21]. Regarding the quality of evidence and risk of bias, our analysis concluded that the selected studies have a low risk of bias, and most are of a high quality of evidence. Regarding those articles funded by the industry or whose authors stated some conflict of interest, the bias risk was well controlled during the peer-review process and did not invalidate the published results.

Two articles based on data of asthmatic patients treated with omalizumab were selected. Both extracted data from a dataset publicly available in Gene Expression Omnibus (GEO) (https://www.ncbi.nlm.nih. gov/geo/; checked on 1 November 2021), i.e., GSE134544, that was uploaded by Upchurch et al. [18]. This particular dataset is of great interest since it includes gene expression profiles from responders and non-responders to omalizumab treatment. CD3E was identified as a suitable biomarker for evaluating response to therapy since an increase in its expression was observed in responders compared with non-responders. CD3E is expressed on the surface of T cells and plays an essential role in T cell development and activation [19]. Another potential response biomarker raised from this study was Galectin-3. This protein binds to IgE impeding the formation of the complex IgE-FcεRI, and therefore, reinforcing the action of omalizumab [35]. The contributing authors, Upchurch et al., performed a clustering analysis of global gene signatures of responders and non-responders to omalizumab, finding eight clusters of genes involved in protein synthesis, T cell regulation and activation, and inflammation, among others. Interestingly, both responders and non-responders showed signature differences between baseline and first weeks of omalizumab treatment. However, these differences disappeared in non-responders by the end of the monitoring time (week 26), while responders exhibit significant differences between 0 and 26 weeks, being more similar to healthy controls at the final time point [18].

Current treatment guidelines for patients with severe, uncontrolled asthma with eosinophilia recommend anti-IL-5 therapy [1]. The mechanism of response to these anti-IL-5 antibodies, i.e., mepolizumab and reslizumab, has been mainly attributed to inhibition of IL-5 response on eosinophils. However, a recent study using dexpramipexole, which completely depletes all eosinophils, failed to show any significant improvement of symptoms [36], suggesting that eosinophils are not the only effector cells, and other cell types may also be involved. Also, targeting the IL-5 may not completely deplete eosinophils, leading to a poor response to therapy. Conversely, anti-IL-5Rα (benralizumab) rapidly depleted eosinophils and significantly reduced the rate of exacerbations for patients with uncontrolled eosinophilic asthma [37]. 

Two studies focused on mepolizumab therapy, showing contradictory results. The earlier one reported no genetic changes upon mepolizumab treatment on severe asthma patients [21]. At the same time, the latter described a reduction in inflammatory mediators, such as prostaglandins (PG) D2 and F2α, leukotrienes E4 and B4, and thromboxane B2 when comparing mepolizumab-treated AERD patients with AERD patients upon other treatments [20]. Also, mepolizumab proved to increase the expression of the PGD2 receptor (CRTH2) on the surface of eosinophils and basophils, although total counts were reduced. Since these two studies were performed in patients with different pathologies, it is likely that AERD patient gene expression was differentially affected by the treatment, as the AERD gene expression profile is known to diverge from that of aspirin-tolerant asthmatics [38]. 

Rial et al. compared both anti-IL-5 antibodies’ effects on gene expression in two groups of severe eosinophilic patients [28]. They found differences in miR-338-3p between the baseline and eight weeks of treatment, although both biologicals got the same effect, at least concerning this miRNA. That study introduces epigenetic granges as involved in response to biologics. Further comparative studies between mepolizumab and reslizumab could reveal differences that facilitate the assignment of patients to one or the other anti-IL5 treatment.

While mepolizumab and reslizumab target the same molecule and are likely to behave similarly, evaluation of anti-IL-5 (mepolizumab) and anti-IL-5Rα (benralizumab) in parallel may raise significant differences in gene expression. A couple of studies conducted by the same group compared serum proteomics of a severe asthmatic treated with mepolizumab or benralizumab and healthy controls [26,27]. When comparing the baseline with one month of treatment, an increase in ceruloplasmin was seen in the benralizumab-treated group but not in the mepolizumab-treated patients. Ceruloplasmin is a ferroxidase enzyme that forms free radicals [39], contributing to the antioxidant effect of treatment. The authors confirmed this result in a later article, and proposed ceruloplasmin as a potential biomarker for monitoring benralizumab treatment. 

Besides ceruloplasmin, other potential biomarkers of response to benralizumab have been proposed in the reviewed articles. Thus, Nakajima et al. [24] identified four transcriptional clusters in blood from severe asthmatics, cluster 2 being the one that agglutinated most of the super-responders. These patients had the highest numbers of eosinophils, higher numbers of basophils, and higher expressions of genes related to eosinophil activities. Conversely, cluster 1 included poor responders to benralizumab. It was characterized by the upregulation of genes related to neutrophils, such as *OLFM4*, which is produced by neutrophils and has been associated with asthma inflammation [40], and CTSG, the neutrophil protease cathepsin G, which has been involved in neutrophilic asthma [41]. In this sense, it has been described that increased sputum neutrophils can be associated with exacerbations in patients treated with benralizumab [42]. Sridhar et al. also reported a significant reduction in the eosinophil signature upon benralizumab treatment, mainly in genes, such as *CLC*, *OLIG2*, and *FCER1A* [25]. CLCs are known as a classical hallmark of eosinophilic inflammation [43], OLIG2 is expressed in eosinophils and associated with the control of *SIGLEC* 8 expression [44], and FcεR1A (*FCER1A*) is the high affinity receptor for IgE and expressed on eosinophils and basophils [45].

Benralizumab treatment seemed to alter the expression level of genes and miRNAs related to the PI3K/Akt signaling pathway [23], which is known to have a regulatory role in allergic asthma [46]. Also, the inactivation of this pathway could modify the response to steroids, supporting the reduction of oral corticosteroid dosage observed in benralizumab-treated patients [33]. Other miRNAs have been proposed as biomarkers of response to treatment, i.e., miR-1246, miR-5100, and miR-338-3p [22], opening a new window of monitoring of the patient evolution.

Finally, we would like to highlight an article that used a completely different approach. Taking advantage of the use of anti-IL-22 (fezakinumab) in atopic dermatitis patients, Badi et al. built an FZ-response gene expression signature and evaluated whether it could be identified in severe asthmatic patients [29]. IL-22 is involved in atopic dermatitis and may be relevant in the atopic march [47]. Interestingly, they found that the FZ-response signature was enriched in neutrophilic (low T2) asthma patients, and therefore, they could benefit from fezakinumab treatment. It is worth noting that to date, there is no approved biologic for T2-low asthma [33], so this approach may open a new opportunity for these patients.

The present systematic review has been conducted following GRADE recommendations. A thorough search of published data yielded over 100 articles, but only 12 met the criteria we set for analyzing the effects of therapeutic monoclonal antibodies for severe asthma on gene/protein expression at the genome- or proteome-wide level. The scarcity of studies addressing this topic is a limitation of the present review, but we have to consider that most treatments have been used in real settings for just a few years and further clinical trials are expected in the short term (NCT04565483, NCT04641741, NCT03476109). Therefore, our review may contribute to setting the criteria and outcomes for these potential future trials. Regarding the current studies, our main concerns relate to the links with the industry of some of the authors that could bias the published results. In fact, only one out of the 12 articles reported negative results, finding no genetic association with mepolizumab efficacy.

On the other hand, this review has some strengths we would like to highlight. Our main goal was to provide clinicians with some potential biomarkers of efficacy that could contribute to better monitoring of biological therapy against severe asthma. By comparing the approved treatments and tabulating the main outcomes, we offer a comprehensive overlook of the state-of-the-art knowledge on these treatments for severe asthma from the molecular point of view. We have also included clustering information that may help stratify patients and select the best treatment for each group. Finally, we included a study that explored an innovative strategy by searching for gene signatures of atopic dermatitis in severe asthmatic, trying to identify who could benefit from anti-IL-22 antibody treatment. Thus, the comparison between genetic profiles of responders to other therapies in related diseases and those of severe asthmatics might discover new applications for already approved biologicals.

## 5. Conclusions

Although limited, data about changes on genomic and proteomic upon biological treatments for asthma published to date are very promising and may set the path for the use of biomarkers in response to these therapeutic antibodies. New trials that go deeper into the subject are mandatory to contrast and validate the current information, and some clinical trials aiming at studying the effects of benralizumab, omalizumab, and mepolizumab on transcriptome and proteome of patients are currently ongoing. Studies focused on the molecular aspects will be conducted and published in the coming years, as more and more patients benefit from this type of treatment. Multicenter, multiethnic, multiage trials including such a perspective would provide comprehensive information about the effects of biological therapies in a diverse population, allowing for a more accurate clustering of patients according to their molecular background. Strict inclusion criteria, exhaustive clinical characterization of patients, and best procedural and analytical practices will permit comparison between treatments, which stands out as a requirement for the efficient and cost-effective management of severe asthma.

## Figures and Tables

**Table 1:** Summary of selected studies.

Ref.	Biologic Therapy	Study Type	Disease	Objective/s	Sample Size	Time of Treatment	Main Results
Zhang et al., 2021	OMA	Transcriptome	Severe asthma	To identify the biomarkers for predicting treatment response to omalizumab	45 patients 11 NRs 34 Rs 17 HC	0, 6, 14, and 26 weeks	A gene module (547 genes) predominated in responders. *CD3E*, a predictive biomarker for response. Other potential biomarkers: *CD79*, serum periostin, galectin 5, *CXCL10*, and *IL*-12
Upchurch et al., 2020	OMA	Transcriptome	Moderate to severe asthma	To investigate the transcriptional variations between responders and non-responders; to study the mechanisms of action	45 patients 34 Rs 11 NRs 17 HC	0, 6, 14, and 26 weeks	Eight gene clusters identified. Upregulation of neutrophil and eosinophil activities in NRs, independent of treatment. Gene expression in responders, more similar to that of HC after treatment.
Condreay et al., 2017	MEP	GWAS	Severe asthma	To investigate genetic associations that may predict response to treatment	148 placebos 441 MEP	1 year	Eight gene variants had weak evidence of association with treatment. No genetic marker was significantly associated with exacerbation rate.
Buchheit et al., 2021	MEP	Single-cell RNA sequencing	AERD	To identify the mechanisms by treatment improves respiratory inflammation	36 AERD patients: 18 MEP 18 other	3 months	Decreased production of inflammatory eicosanoids. Upregulation of genes involved in tight junction pathways (*TJP3*, *ACTN4*, and *AMOT*) and cilium organization.
Landi et al., 2017	MEP BEN	Proteomics	SEA	To compare the serum proteomic profiles, before and after one month of therapy for molecular modifications	10 patients MEP 8 patients BEN 4 HC	Baseline, 1 month	Benralizumab: Increased plasmin, α-1-antitrypsin, plasminogen, α-2-macroglobulin, and ceruloplasmin levels. Mepolizumab: increases in albumin, fibrinogen γ, and factor B levels
Vantag-giato et al. 2020	MEP BEN	Proteome	Severe asthma	To compare the serum proteomic profiles of patients before and after treatment	10 patients: 5 MEP 5 BEN	Baseline, 1 month	Ceruloplasmin is a potential biomarker for benralizumab treatment.
Sridhar et al., 2019	BEN	Transcriptome Proteome	Severe asthma and COPD	To investigate the effects of treatment on blood inflammatory markers.	Asthma patients: 395 for proteome 326 for transcriptome COPD patients: 84 for proteome 78 for transcriptome	0, 52 weeks (asthma study) 0, 32 weeks (COPD study)	Benralizumab: upregulation of eotaxin-1 and eotaxin-2. Significant reductions in eosinophil-associated signatures.
Nakajima et al., 2021	BEN	Transcriptome	SEA	To identify gene expression patterns in response to benralizumab; to determine correlation with clinical responsiveness	41 patients	Baseline, 4 months	33 eosinophilic genes and 29 neutrophilic genes (4 clusters) associated with response to treatment
Hirai et al., 2021	BEN	Gene expression analysis (qPCR)	Severe asthma	To elucidate the influence of treatment on key molecules involved in steroid responses	17 patients	0, 4, 8, 16, and 24 weeks 30 mg per dose	Increased expression levels of PI3K-associated genes (*HDAC2*, *NFE2L2*, *GLCCI1*, and *PTEN*). Decreased level of miR-21-5p Inhibition of PI3K pathway.
Cañas et al., 2021	BEN	Gene expression analysis (qPCR)	SEA	To search some miRNAs that could serve as biomarkers to detect an early response	15 SEA patients 15 MA patients	Baseline, 8 weeks	Decreased expression of three miRNAs (miR-1246, miR-5100 and miR-338-3p)
Rial et al., 2021	RES MEP	Gene expression analysis (qPCR)	SEA	To analyze possible changes in serum miRNAs in patients upon treatment	16 patients 10 RES 6 MEP	Baseline, 8 weeks	miR-195-5p and miR-27b-3p were downregulated miR-1260a (*p* < 0.05), miR-193a-5p (*p* < 0.01), and miR-338-3p (*p* < 0.05) were upregulated
Badi et al., 2021	FZ	Transcriptome	Severe asthma	To determine whether the AD transcriptomic signature of responders to fezakinumab (FZ) is enriched in severe asthma patients	421 SA 88 MA 101 HC	12 weeks	The FZ-response signature (296 down-, 144 upregulated genes) was enriched in blood from neutrophilic asthmatic patients

Abbreviations: Ref., reference; NRs, non-responders to treatment; Rs, responders to treatment; HC, healthy controls; GWAS, genome-wide association study; AERD, aspirin-exacerbated respiratory disease; SEA, severe eosinophilic asthma; SA, severe asthma; MA, mild/moderate asthma; COPD, chronic obstructive pulmonary disease; AD, atopic dermatitis; qPCR, quantitative PCR; OMA, omalizumab; MEP, mepolizumab; BEN, benralizumab; RES, reslizumab; FZ, fezakinumab.

**Table 2:** Results of the RoB2 analysis, as per assignment to intervention (the ‘intention-to-treat’ effect). Total number of studies: 12.

	Randomization Process	Deviations from Intended Interventions	Missing Outcome Data	Outcome Measurement	Selection of the Reported Result	Overall Bias
Low risk	100%	100%	100%	100%	100%	100%
Some concerns	-	-	-	-	-	-
High risk	-	-	-	-	-	-

**Table 3:** Evaluation of quality of evidence, as per the appraisal form for Longitudinal Studies by the Evidence Evaluation Tools and Resources (LEGEND). Articles are identified by their numbers in the Reference List below. Red circles indicate some concerns in the area evaluated. Total score was obtained from the number of green circles. High: 9–11; Moderate: 6–8.

Reference Number	Adequate Aim/Criteria	Validity					Reliability				Applicability	Assessment
		Appropriate Methods	Appropriate Technology	Clearly Described Methodology	Clearly Described Outcomes	Conflict of Interest	Appropriate Statistical Analysis	Sample Size	Precision	Significant Results		
18	●	●	●	●	●	●	●	●	●	●	●	High
19	●	●	●	●	●	●	●	●	●	●	●	High
20	●	●	●	●	●	●	●	●	●	●	●	High
21	●	●	●	●	●	●	●	●	●	●	●	High
22	●	●	●	●	●	●	●	●	●	●	●	High
23	●	●	●	●	●	●	●	●	●	●	●	High
24	●	●	●	●	●	●	●	●	●	●	●	High
25	●	●	●	●	●	●	●	●	●	●	●	High
26	●	●	●	●	●	●	●	●	●	●	●	Moderate
27	●	●	●	●	●	●	●	●	●	●	●	Moderate
28	●	●	●	●	●	●	●	●	●	●	●	Moderate
29	●	●	●	●	●	●	●	●	●	●	●	High

**Table 4:** Summary of potential biomarkers of response to treatment. All molecules/pathways are upregulated upon treatment unless otherwise indicated (↓).

Treatment	Genes/miRNAs	Proteins	Pathways
Omalizumab	*CD3E*		Th2 response (*CSF3*, *IL4*, *IL5*, *IL18* and *SPI1*)
	*CD79*		Th1 response (*STAT1*, *STAT4*, *IL2* and *SMARCR4*)
			↓ Suppression of inflammation (*TWIST1*, *FOXO1*, *FOXO3*, *TP53*, *CTNNB1*, and *SIM 1*)
Benralizumab	↓miR-21-5p	plasmin	PI3K-associated genes (*HDAC2*, *NFE2L2*, *GLCCI1*, and *PTEN*)
	↓miR-1246	α-1antitrypsin	
	↓miR-5100	plasminogen α-2 macroglobulin	
	↓miR-338-3p	ceruloplasmin	
		eotaxin-1	
		eotaxin-2	
Mepolizumab	↓miR-195-5p		Tight junction function (*TJP3*, *ACTN4*, and *AMOT*)
	↓miR-27b-3p		
	miR-1260a		
	miR-193a-5p		
	miR-338-3p		

## Data Availability

The data presented in this study are available on request from the corresponding author. The data are not publicy available due to privacy.

## Supplementary Materials

The following supporting information can be downloaded at: https://www.mdpi.com/article/10.3390/biomedicines10020293/s1, Table S1: PRISMA 2020 Checklist.
